# Tensins in Kidney Function and Diseases

**DOI:** 10.3390/life13061244

**Published:** 2023-05-24

**Authors:** Chien-Wei Huang, Su Hao Lo

**Affiliations:** 1 Department of Biochemistry and Molecular Medicine, School of Medicine, University of California-Davis, Sacramento, CA 95817, USA; 2 Division of Nephrology, Department of Internal Medicine, Kaohsiung Veterans General Hospital, Kaohsiung 81362, Taiwan; 3 School of Medicine, National Yang Ming Chiao Tung University, Taipei 112304, Taiwan

**Keywords:** tensin, CTEN, focal adhesion, cystic kidney, nephrotic syndrome, cancer

## Abstract

Tensins are focal adhesion proteins that regulate various biological processes, such as mechanical sensing, cell adhesion, migration, invasion, and proliferation, through their multiple binding activities that transduce critical signals across the plasma membrane. When these molecular interactions and/or mediated signaling are disrupted, cellular activities and tissue functions are compromised, leading to disease development. Here, we focus on the significance of the tensin family in renal function and diseases. The expression pattern of each tensin in the kidney, their roles in chronic kidney diseases, renal cell carcinoma, and their potentials as prognostic markers and/or therapeutic targets are discussed in this review.

## 1. Introduction

Tensins are cytoplasmic phosphoproteins localized to focal adhesions (FAs) that are transmembrane junctions between the extracellular matrix (ECM) and intracellular cytoskeletal networks, mainly through integrin transmembrane receptors and various FA proteins. FAs serve as structural complexes mediating bidirectional communications across the cell membrane [1]. Tensins provide one such type of molecular linkage by interacting with the cytoplasmatic tails of ß integrins and actin filaments via their phosphotyrosine-binding (PTB) domains and actin-binding domains (ABDs), respectively [2,3]. Besides their structural roles, tensins transduce numerous signaling pathways, such as the AMPK, Rho GTPase, Mek/Erk, Src/Fak, and PI3K/Akt/mTOR pathways. These and other pathways contribute to tensins’ regulatory roles in cell adhesion, migration, invasion, polarity, proliferation, and mechanical sensing [4,5,6,7,8,9].

**Figure 1:**
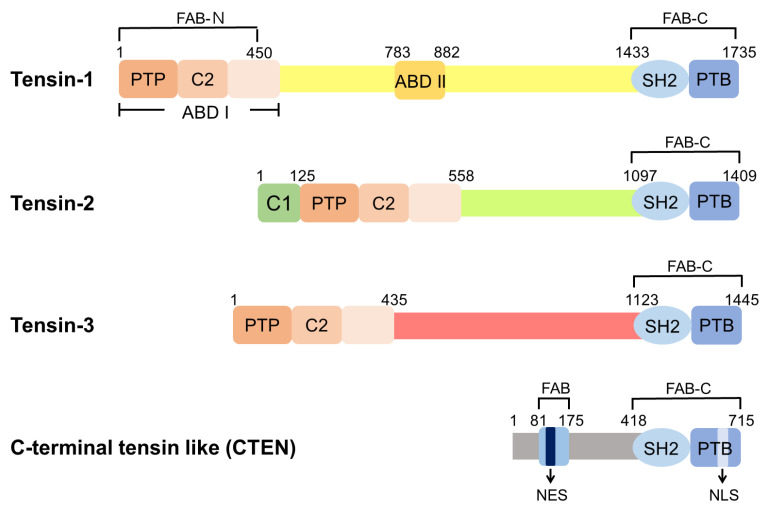
Domain structures of human tensins. Tensin-1 (TNS1), tensin-2 (TNS2), and tensin-3 (TNS3) have similar domain structures, including the PTP, C2, SH2, PTB, ABD, and FAB domains. There are two independent FAB regions: FAB-N domains contain the PTP and C2 domains at the N-terminal region and FAB-C overlaps with the SH2 and PTB domains at the C-terminus. ABD I, which interacts directly with actin filaments, is located at the N-terminus and overlaps with FAB-N. ABD II binds to the barbed end of the actin filaments. The protein kinase C conserved region 1 domain (C1 domain) is found in TNS2 with an uncertain function. CTEN harbors the SH2-PTB tandem domain and contains the FAB-C domain. Although CTEN also features a second FAB domain in its N-terminal region, its amino acid sequence is different from those of FAB-N in other tensins [14]. Moreover, there is a nuclear export sequence (NES) localized within this unique FAB site of CTEN and a nuclear localization sequence (NLS) within CTEN’s PTB domain [14].

There are four members in the mammalian tensin family, namely tensin-1 (TNS1), tensin-2 (TNS2), tensin-3 (TNS3), and C-terminal tensin-like (CTEN, also known as tensin-4, TNS4) (Figure 1). TNS1, TNS2, and TNS3 are larger proteins of 170–220 kDa and share similar domain structures, comprising the protein tyrosine phosphatase (PTP), C2, Src homology 2 (SH2), PTB, ABD, and focal-adhesion-binding (FAB) domains [10,11,12]. CTEN is a smaller protein of ~80 kDa and harbors the SH2-PTB tandem domain and the FAB-C domain, similar to other tensins [13]. These protein structures suggest that tensins may share similar binding/biological activities, while each features its specialized function.

The potential associations of tensins with human diseases were reported using analysis of genome-wide association studies. These studies showed that variants in *TNS1* were associated with high risks of mitral valve prolapse, chronic obstructive pulmonary disease, and asthma with hay fever phenotype [15,16,17,18]. Abnormalities in heart valves were confirmed in animal models, including atrioventricular valve regurgitation in *Tns1*-knockdown zebrafish and enlarged posterior mitral leaflets in *Tns1*-knockout (KO) mice [15]. The association between tensins and cancers have been evaluated, mainly through expression profiling, in numerous studies [19,20,21,22,23,24,25,26,27,28,29,30,31]. Among them, upregulation of CTEN was reported to be associated with poor prognosis in patients with colorectal cancer, lung adenocarcinoma, breast cancer, gastric cancer, hepatocellular carcinoma, and melanoma [22,23,24,25,26,27,28,29,30,32,33]. Nonetheless, the impacts of tensin expressions on cancer prognosis are not always consistent. For example, upregulated TNS1 levels increased the risk of mortality in patients with colorectal cancer but were associated with better survival in patients with lung adenocarcinoma [9,34,35,36]. These findings suggest that the impacts of tensins on the prognosis of malignancy depend on the types of tensins and cancers.

The first sign showing the association between tensins and kidney diseases was from the study of *Tns1*-KO mice [37]. Mice lacking TNS1 developed chronic kidney disease (CKD) with cystic formation. The genetic study demonstrated that *Tns2* was the disease-causing gene in the ICR-derived glomerulonephritis (ICGN) mouse line with a hereditary nephrotic syndrome (NS) [38] and their symptoms were dedicated by mouse genetic backgrounds [39]. The relevance of these mouse studies with human diseases is supported by the downregulation of TNS1 levels in human patients with polycystic kidney disease, as well as the identification of *TNS2* mutations in NS patients [40,41]. Considering the potential of tensins as candidates for precision medicine, a comprehensive review of tensins in kidney function and diseases is highly warranted. In the current review, we present an overview of tensin expressions in the kidney and provide current evidence supporting the association between each tensin and CKD and kidney cancer, as well as the clinical implications of these findings. 

## 2. Tensin Expressions in the Kidney

*TNS1-3* mRNAs are readily detected in human kidneys by Northern blotting, whereas *CTEN* signaling is extremely weak [10,11,12,13]. This expression pattern is further confirmed using isolated glomeruli and non-glomeruli for reverse transcription-polymerase chain reaction assays showing that *TNS1* transcripts are more abundant in renal tissues, except glomeruli, whereas *TNS3* shows an opposite pattern [42]. Meanwhile, *TNS2* mRNA levels are similar between glomeruli and non-glomeruli in the kidney, and *CTEN* is not detectable [42]. At the protein level, TNS1 is most concentrated at the proximal and distal tubules in the cortex region, as well as Bowman’s capsules and mesangial cells in glomeruli by immunohistochemistry staining [37,42,43]. Immunohistochemistry staining shows that TNS2 proteins are expressed at the podocytes, mesangial cells, tubules, collecting ducts, and renal pelvis epithelia, whereas TNS3 proteins are present at the podocytes, tubules, collecting ducts, and renal pelvis epithelia [42]. By immunoelectron microscopy, anti-TNS1-labelled gold particles were detected periodically along the basolateral membranes of tubular epithelial cells and the membranes of endothelial cells. Interestingly, both small and large clusters of gold particles are observed, suggesting at least two types of TNS1-positive focal adhesion complexes in kidney epithelial cells [37]. The expression patterns of tensins provide hints regarding their potential roles in the kidney.

## 3. Tensins in Chronic Kidney Diseases

### 3.1. TNS1

**Figure 2:**
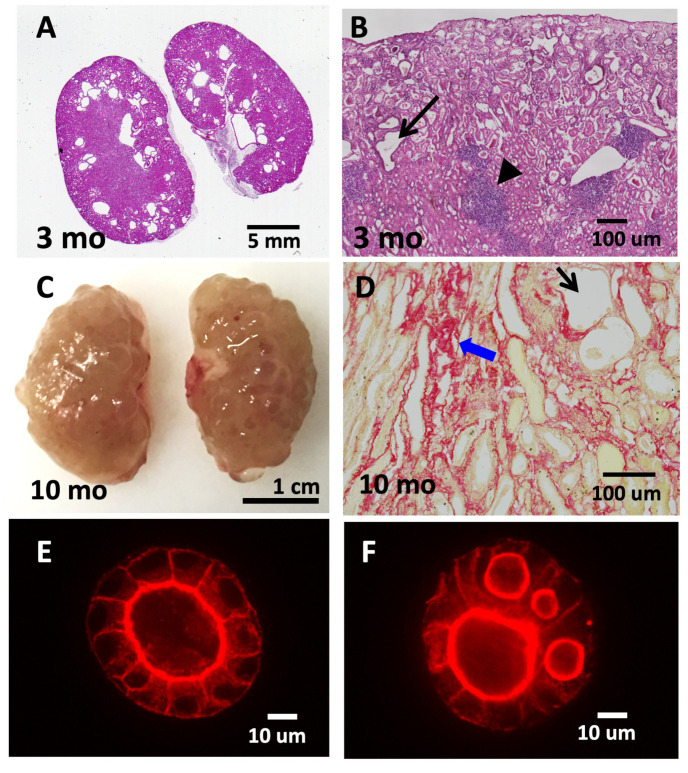
*Tns1*-KO mice develop features of cystic kidney disease. *Tns1*-KO kidneys isolated from 3-month-old (**A**,**B**) or 10-month-old (**C**,**D**) mice were processed for H&E staining (**A**,**B**), Sirius Red staining (**D**), or general morphology (**C**), showing dilated tubules (black arrows), interstitial infiltration (arrowhead), fibrosis (blue arrows), and cystic kidneys. Representative images of *Tns1* WT (**E**) or KO (**F**) MDCK in 3D Matrigel culture for 5 days and stained for actin.

The involvement of TNS1 in kidney function was first observed in *Tns1*-KO mice [37]. These mutant mice are completely developed, and their kidneys are clinically and histologically normal for few months. Nonetheless, signs of renal tubular dilatations, interstitial infiltrates, and fibrosis are observed around 3 months of age (Figure 2). The renal condition progressively deteriorates, and mice usually die within 16 months. Proteinuria is only detected near the end stage of life. Cysts are only found in the kidneys and not in other organs. The renal phenotype observed in *Tns1*-KO mice has been suggested as one of the animal models of cystic kidney disease showing genetic recessive inheritance and renal defect onset at adulthood [44]. These features are quite different from autosomal dominant polycystic kidney disease (ADPKD), which is caused by one copy of the affected gene, and autosomal recessive polycystic kidney disease (ARPKD), which usually shows severe symptoms during infanthood or childhood. Nonetheless, a recent study revealed that the protein expression of TNS1 was significantly decreased in human ADPKD tissue than in normal kidney tissue, indicating the potential role of TNS1 in the development of cysts in patients with ADPKD [40]. Accumulating studies have suggested that, in spite of differences in the causal gene, age of onset, disease severity, and cyst distribution of various cystic kidney diseases, cyst formation appears to commonly arise from dysregulated cell growth or death, increased secretion into the tubular lumen, aberrant cell–matrix or cell–cell interaction loss of cellular polarity, or cilium dysfunction [45]. Therefore, analyses of mutant mice with cystic kidneys, such as *Tns1*-KO mice, contributes to a deeper understanding of and better treatments for a variety of cystic kidney diseases.

When kidney epithelial cells, such as Madin–Darby canine kidney (MDCK) cells, are grown in a three-dimensional (3D) extracellular-matrix (ECM) environment, the suspended cells migrate, proliferate, polarize, and expand to form hollow, fluid-filled spherical monolayers, which are usually called cysts or spheres. When these spheres are incubated with hepatocyte growth factor, a subset of cells from the monolayer wall protrudes into the ECM and further develops into a tube. This in vitro 3D model recapitulates many features of in vivo renal tubules and provides a powerful system to study the development and molecular mechanisms in a way that readily allows experimental manipulation under relatively physiological conditions. Three general principles are required for cells to form a lumen in 3D culture: cell–cell and cell–matrix recognition; establishment of apical–basal polarity; and lumen expansion. Many critical steps and molecules have been established using this MDCK 3D system [46,47,48]. With this in mind, a *Tns1*-KO MDCK 3D cell-culture system was established to investigate molecular mechanisms related to cystic formation [8]. Instead of forming cysts with a single lumen as in wild-type (WT) MDCK cells, *Tns1*-KO cysts contain multiple lumens (Figure 2) [8]. Further analyses show that *Tns1*-KO cells are capable of establishing apical–basal polarity and the cell–cell junction, and they form the apical membrane initiation site (AMIS); however, AMIS in *Tns1*-KO cells develop much faster than WT cells and cannot ensure only one AMIS is formed in each cyst [8]. At the molecular level, Mek/Erk activities are upregulated in *Tns1*-KO 3D cells and Mek-inhibitor treatments significantly convert multiple lumens to a single lumen. In agreement with the in vitro findings, Mek/Erk activities are markedly increased in the *Tns1*-KO over the WT mouse kidneys. Treated with a Mek inhibitor such as trametinib, pMek and pErk levels are reduced, and signs of interstitial infiltrates, fibrosis, and dilated tubules are significantly improved in *Tns1*-KO mouse kidneys [8]. This study provides a potential therapeutic strategy using Mek inhibitors for cystic kidney diseases. Furthermore, TNS1 proteins phase-separate to biomolecular condensates, a type of membrane-less organelle, in MDCK cells [49]. The presence of TNS1 condensates is dependent on the cell cycle, and TNS1 condensates contain particular focal adhesion proteins and signaling molecules, such as pT308Akt. These results suggest that TNS1 condensates are involved in the disassembly of FAs, the storage of core FA components, and the formation of signaling intermediates. However, the impact of TNS1 condensates on renal function and disease requires further investigation.

Although no human cystic kidney patient associated with *TNS1* mutations has been reported so far, the likelihood is very high based on the finding that *Tns1*-KO mice are able to produce offspring, develop renal defects progressively, and have half the normal life expectancy. It is highly possible that a subgroup of human patients with cystic kidney diseases is associated with *TNS1* aberrant expressions and/or mutations. These patients may also be associated with abnormal mitral valves and wound-healing processes as observed in *Tns1*-KO mice [15], as well as chronic obstructive pulmonary disease, which *TNS1* has been identified as a high-risk gene for through genome-wide association studies [15,16,50].

### 3.2. TNS2

**Figure 3:**
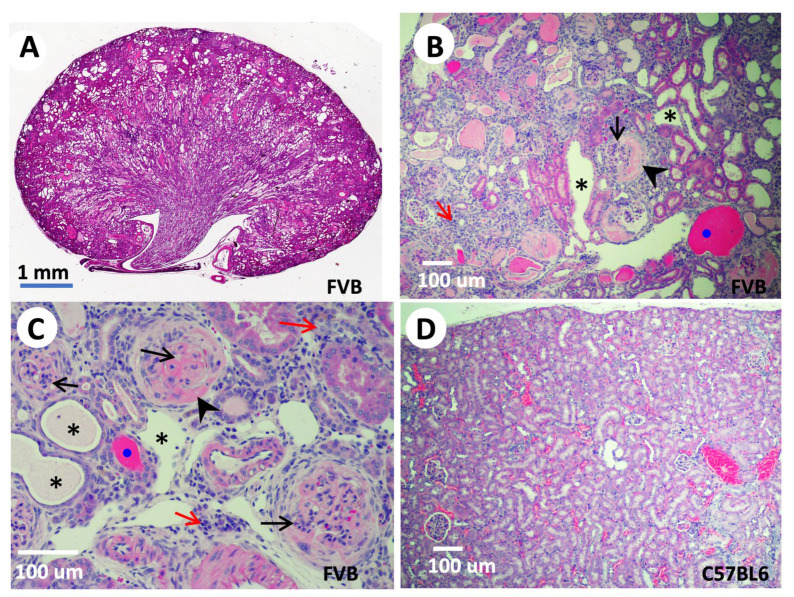
*Tns2*-KO FVB mice develop features of glomerulonephritis. Kidneys isolated from 9-week-old (**A**) or 6-week-old (**B**,**C**) *Tns2*-KO FBV mice, generated in the laboratory using *Tns2*-KO embryonic stem cells from the Knockout Mouse Programme (www.mousephenotype.org, accessed on 10 October 2015), were processed and stained with H&E, showing mesangial proliferation (black arrows), segmental glomerulosclerosis (arrowhead), tubular dilatation (asterisk), cast (blue dot), and interstitial infiltration (red arrow). These defects are mouse-strain-dependent and are not developed in the kidneys of *Tns2*-KO C57BL6 mice, even at 2 years of age (**D**).

The first evidence of the kidney relevance of TNS2 came from genetic analysis of ICGN mice, a spontaneous mutant strain with a hereditary NS displaying glomerulosclerosis, vascular sclerosis, and tubulointerstitial fibrosis histologically, as well as proteinuria and anemia clinically [51]. These mice often die within 26 weeks of age [52]. NS is a common cause of human CKD that may result in end-stage renal disease requiring dialysis or renal transplantation. At the molecular level, excessive accumulation of ECM components, including laminin, collagen, and fibronectin [53], decreased matrix metalloproteinase activity [54], and abnormal expressions of integrins in glomeruli [55] were found in ICGN kidneys. Through quantitative trait locus analysis using albuminuria as a tracking symptom of NS, the main cause of gene was mapped to a region on mouse chromosome 15. Direct sequencing of the coding regions of candidate genes within the mapped area identified a deletion of eight nucleotides in exon 18 of the ICGN *Tns2* gene [38]. This deletion not only causes a frameshift leading to early termination, but also results in a decreased transcript level and lack of TNS2 protein expression in ICGN mice [38]. Intriguingly, when these mutant mice were backcrossed to either C57BL6 [39] or sv129 strains [56], the *Tns2* mutant mice showed no sign of NS or any renal defects. On the other hand, *Tns2* mutant mice in FVB or DBA [57,58,59,60] display s thickened glomerular basement membrane, the effacement of podocyte foot processes, mesangial proliferation, dilated tubules, casted cysts, interstitial inflammation, and massive proteinuria, as expected (Figure 3). These findings indicate that NS phenotypes caused by lack of TNS2 are dictated by genetic modifiers. Through quantitative trait locus, congenic, and genome-wide linkage analyses, a proximal region on chromosome 2 associated with tubulointerstitial fibrosis, a distal region on chromosome 2, and a region on chromosome 10 associated with podocyte damage induced by *Tns2* mutations were identified [61,62]. The identities of these phenotypic resistant modifiers remain to be revealed. The generation of *Tns2* mutant FVB mice expressing a truncated TNS2 lacking its SH2 and PTB domains not only provides direct proof of the absolute requirement of TNS2, but also demonstrates the essential roles of SH2 and PTB domains in maintaining podocyte integrity and function [58]. The study suggests that impaired mechanical adjustment to biomechanical stress due to TNS2 deficiency contributes to podocytopathy and NS in mice [63]. In contrast, mutant mice only expressing inactive mutant TNS2 C231S PTPase maintain normal renal structure and function [64], indicating that the PTPase activity of TNS2 is not required for renal function maintenance. 

Interestingly, it has been reported that TNS2 regulates podocyte hypertrophy through mTORC1 activation [6], which is known to impair podocyte function in diabetic nephropathy [65]. It is reported that upregulated TNS2 dephosphorylates pY-nephrin and disrupts the pY-nephrin/PI3K complex via TNS2’s PTPase activity. In turn, it promotes the pY-IRS1/PI3K complex that activates the mTORC1 pathway [6], and mTORC1 hyperactivation leads to podocyte hypertrophy. Overexpression of TNS2, but not the C231S mutant, in podocytes increases cell sizes and albumin leakage in permeability assays. Similarly, mice injected with adenovirus carrying *Tns2*, but not the C231S mutant, into the kidneys show higher levels of pY-mTORC1, show reduced levels of pY-nephrin, and develop signs of NS, including albuminuria and podocyte injury [6]. These studies indicate that overexpression of TNS2 in the kidney also induces NS and that the PTPase activity of TNS2 is essential. Nonetheless, as mentioned earlier, a recent report showed that mutant mice only expressing the TNS2 C231S mutant were normal without NS [6]. The role of TNS2’s PTPase activity in renal structure and function requires further clarification.

The direct link of TNS2 to human nephrotic syndrome came from the identification of *TNS2* mutations in four families with partially treatment-sensitive NS [41]. These patients contain homozygous or compound heterozygous missense mutations of *TNS2* (also called nephrotic syndrome type 19, NPHS19), strongly suggesting recessive *TNS2* mutations as a novel cause of NS [41].

### 3.3. TNS3

The connection between TNS3 and non-cancer kidney diseases is yet to be explored. The only report slightly related to this found that a chromosomal translocation resulting in *TNS3-EXOC6B* fusion genes might be the cause of a developmentally delayed newborn child who suffered from neutropenia and pulmonary infections and had only one kidney [66]. However, with growing cases showing *EXOC6B* translocations in developmentally delayed patients, *EXOC6B* is more likely to be the major cause of defects [67].

### 3.4. CTEN/Tensin-4

During renal tubulogenesis, several stages are established based on morphological changes in a 2.5-dimensional culturing system [68]. These stages include multicellular apical protrusion, extension, tubule initiation, and tubule elongation. *CTEN* was identified as one of top upregulated genes in the extension stage. Downregulation of CTEN decreases the formation of extensions and tubules, whereas CTEN overexpression promotes cell invasion. However, overexpression of the CTEN SH2-inactive mutant blocks the process at the extension stage and accumulates a higher level of active Stat3, indicating a critical role of the CTEN-Stat3 axis in renal tubulogenesis. The relevance of CTEN in kidney development and function remains to be investigated in animal models.

CTEN has been predicted to function as a dominant negative inhibitor of TNS1-3 based on its shorter protein sequence that only shares critical SH2 and PTB domains with other tensins. Surprisingly, our recent mouse studies showed that expression of CTEN in *Tns1*-KO mice almost fully rescue all renal phenotypes caused by the lack of TNS1 [69], indicating that CTEN is in fact a smaller version of tensin and that SH2 and PTB domains carry the essential functions of the tensin family in kidney development/function. Because the SH2 and PTB domains of TNS2 are required for preventing NS development in FVB mice [58], it will be interesting to test whether *CTEN*-KI can also rescue *Tns2*-KO kidney phenotypes.

Although CTEN expression eases *Tns1*-KO phenotypes, it cannot fully replace TNS1. Compared with *CTEN*-KI mice, there are still mild defects and a slightly shorter lifespan in *Tns1*-KO/*CTEN*-KI mice [69]. Both TNS1 and CTEN contain a focal adhesion targeting site in their N-terminal regions, albeit no sequence similarity between them. On the other hand, CTEN, instead of TNS1, accumulates at keratin intermediate filaments in response to mechanical forces [70] and is able to shuttle to the nucleus [14]. These differences may contribute to the incomplete rescue by CTEN in *Tns1*-KO mice.

## 4. Chronic-Kidney-Disease-Related Pathways or Biomarkers 

CKD is defined as a decrease in renal function (glomerular filtration rate < 60 mL/min per 1.73 m^2^) or kidney damage, evidenced by pathological abnormalities or markers that last for more than 3 months [71]. The characteristics of CKD include nephron loss, reduced renal regenerative capacity, microvascular damage, metabolic changes, oxidative stress, and inflammation, ultimately resulting in fibrosis and ECM accumulation [72]. Of these, metabolic changes such as lipotoxicity and oxidative stress are believed to be the major driving forces for the loss of nephrons, while injured renal resident cells and recruited inflammatory cells promote further inflammation, which activate myofibroblasts and result in fibrosis and ECM accumulation [73]. The major signaling mechanisms involved in the process of CKD include NLRP3 inflammasome, MAPK, PI3K/Akt, and RAAS signaling in lipotoxicity; mTORC, MAPK, PKB, and NF-κB signaling in oxidative stress; NF-κB, NLRP3 inflammasome, JAK-STAT, Toll-like receptor, and cGAS-STING in inflammation; and TGF-β, Wnt, RAAS, and Notch signaling in myofibroblast activation and ECM accumulation [73].

As mentioned earlier and elsewhere [9,74], tensins regulate a variety of signaling pathways. Some are directly involved in CKD, such as the Mek/Erk and PI3K/Akt/mTORC1 signaling pathways [6,8], indicating that tensins could be involved in lipotoxicity, oxidative stress, and apoptosis during the process of CKD. Other tensin-mediated pathways in response to the ECM, including AMPK, Rho GTPase, and Src/FAK may also contribute to the development of fibrosis and the ECM accumulation of CKD [9,74]. The presence of TNS1 biomolecular condensates in kidney cells further raises the potential impact of TNS condensates in renal function and CKD formation [8,49].

In addition to tensins, several potential biomarkers for early CKD detection or prognosis have been reported. These include serum B2M, BTP, and klotho, as well as urinary BTP and klotho for renal function and prognosis [75].

## 5. Tensins in Renal Cell Carcinoma 

Tensins mediate numerous signaling pathways, as well as cytoskeleton remodeling, which are critical for cell adhesion, migration, polarity, and proliferation/apoptosis [9,74]. Dysregulation of these important activities contributes to tumorigenesis. In fact, involvements of tensins, especially TNS1 and CTEN, in various cancers have been reported [9,31,33,74]. Tensins are known to act as mechano-sensors and receive cues from changes in ECM rigidity and activate intracellular signaling pathways that promote the differentiation of fibroblasts into tumor-associated fibroblasts, as well as cell malignant transformation. For example, in response to increased ECM rigidity, the SH2 domain of TNS1 binds to tyrosine-phosphorylated Hic-5 and elicits a mechano-transduction that leads to the activation of the Rho/ROCK signaling pathway and fiber formation in tumor-associated fibroblasts, which in turn increases ECM stiffness and promotes tumor cell proliferation, invasion, and metastasis [76]. It has been reported that upregulated TNS1 accelerates colorectal cancer cellular metastasis [77]. On the other hand, TNS1 promotes p53 expression, thereby inhibiting the proliferation, migration, and invasion of lung adenocarcinoma cells [78]. Therefore, the impacts of abnormal expression of tensins are cancer-type-dependent, as shown in numerous reports [9,31,33,74]. However, studies of tensins in human renal cell carcinoma (RCC) are very limited. These include all four tensin RNAs that are downregulated in RCC [9,79], and promoter hypermethylation contributes to downregulation of TNS3 in RCC [80].

**Figure 4:**
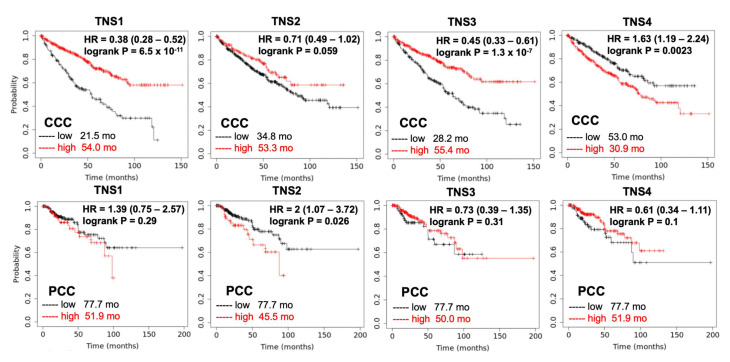
Cumulative survival rate of patients with RCC by tensin status. The Kaplan–Meier curves reveal the cumulative survival rate among patients with CCC (N = 530) and those with PCC (N = 288), stratified according to the status of each tensin. The *p* values are the result of the log-rank test for the comparison between the two groups. High *CTEN* (HR = 1.63, *p* = 0.0023), low *TNS1* (HR = 0.38, *p* < 0.001), and low *TNS3* (HR = 0.45, *p* < 0.001) increase the risk of mortality significantly in patients with CCC. High *TNS2* is significantly associated with poor survival in those with PCC (HR = 2, *p* = 0.026). The association between *TNS1*, *TNS3*, and *TNS4* and survival in patients with PCC is not significant.

Although there are several potential biomarkers for renal cell carcinoma diagnosis or prognosis, such as Cathepsin D and peptide panel [81], no validated RCC prognostic biomarker is currently used in clinical patient care. To test whether expressions of tensins are relevant to the prognosis of patients with RCC, the cumulative survival rate of patients with CCC (N = 530) and PCC (N = 288), using databases collected from KMPlot (kmplot.com, accessed on 15 July 2022), were analyzed according to tensin status (Figure 4). Among four tensins, high *CTEN*, low *TNS1*, and low *TNS3* are highly associated with poor survival of CCC. High *TNS2* has a trend in better survival of CCC, yet it is associated with poor survival in PCC. There is no statistically significant association between *TNS1*, *TNS3*, and *TNS4* and survival in patients with PCC. These pilot analyses suggest that tensins could be potential prognostic biomarkers for PCC and CCC, respectively, and certainly warrant extensive investigations.

## 6. Conclusions

Emerging evidence has demonstrated the critical roles of tensins, especially TNS1 and TNS2, in maintaining normal renal function. Deficiencies in TNS1 or TNS2 result in CKD displaying cystic kidneys or NS, respectively. Recent results have begun to reveal molecular pathways associated with the pathogeneses of these defects, including the Mek/Erk axis in *Tns1*-KO mice and the PI3K/Akt/mTORC1 pathway in *Tns2* mutant mice. The expression pattens and survival analyses of tensins in patients with RCC suggest they have promising values as prognostic markers and even direct targets for RCC. *Tns1*- and *Tns2*-KO cells and mice are excellent systems for drug testing. For instance, Trametinib, a Mek inhibitor, reduces the renal disease burden in *Tns1*-KO mice [8] and dihydro-CDDO-trifluoroethyl amide, a bardoxolone methyl analog, suppresses tubular epithelial cell injury, chronic inflammation, and fibrosis, but not glomerular defects in ICGN mice with *Tns2* mutation [82]. These findings and tools provide great insights into tensins’ clinical importance and applications in the kidney. Nonetheless, more questions remain to be addressed. For example, the PTPase activity of TNS2 is essential for the suggested PI3K/Akt/mTORC1 pathogenic pathway, yet TNS2 C231S mutant mice with inactive PTPase are normal without NS. What additional or alternative mechanisms, such as AMPK, Rho GTPase, and Src/Fak, are involved in CKD caused by TNS1 and TNS2 mutations/deficiencies? Do aberrant TNS1 expression/mutations lead to cystic kidney diseases in humans? What mouse genetic modifiers of *Tns2* are there and are they also present in the human population? What are the expressions and roles of tensins in human kidney diseases, such as diabetic nephropathy, IgA nephropathy, or lupus nephritis? What are the roles of tensins in the progression of CKD? The signaling mechanisms underlying the disease process of renal cell carcinoma mediated by tensins remain elusive and warrant further investigation. These are a few of many areas that require additional efforts in order to further our knowledge on tensins and, more importantly, human health by preventing and/or slowing down the development of renal diseases.

## Data Availability

All data relevant to the study are included in this article.
